# Relatedness as a contextual catalyst in motor learning: the socially embedded motor learning framework

**DOI:** 10.3389/fpsyg.2026.1810460

**Published:** 2026-05-08

**Authors:** Chang-Ha Im

**Affiliations:** 1 Center for Curriculum and Instruction Studies, Korea University, Seoul, Republic of Korea; 2 Department of Physical Education, Korea University, Seoul, Republic of Korea

**Keywords:** autonomy support, cooperative learning, dyadic practice, motor learning, psychological safety, relatedness satisfaction, self-determination theory, social context

## Abstract

Motor learning research has predominantly modeled the learner as an isolated information processor, yielding robust insights into practice schedules and feedback design while leaving the social conditions of skill acquisition comparatively underexamined. This article applies Self-Determination Theory (SDT) to reframe motor learning as a socially embedded process and advances the hypothesis that relatedness—understood here as the learner’s sense of being cared for, respected, and connected to relevant others—functions as a contextual catalyst shaping how autonomy and competence support translate into learning gains. Three testable propositions are advanced: (a) an amplification hypothesis, predicting that autonomy- and competence-supportive practices yield stronger learning effects when relatedness satisfaction is high; (b) a safety-to-exploration pathway, positing that psychological safety mediates the relation between relatedness satisfaction and adaptive learning behaviors such as error tolerance and strategic variability; and (c) a cooperative-structure advantage, proposing that dyadic and team-based formats outperform individual practice when they support relatedness satisfaction and enable informational dialogue. Boundary conditions are also identified—task complexity, expertise level, cultural context, and evaluation pressure—under which these effects may vary, attenuate, or reverse. These ideas are integrated into the Socially Embedded Motor Learning (SEML) framework, together with a methodological agenda emphasizing multi-session designs, multilevel analysis, and psychophysiological or neurocognitive measurement. Practical implications for coaches, physical educators, and rehabilitation professionals are also outlined. By making the social dimension of motor learning theoretically explicit and empirically testable, the SEML framework offers a research program linking motivational psychology, motor control, and pedagogy.

## Introduction

For more than four decades, motor learning research has operated within what might be called an isolated-learner paradigm: the performer is modeled as an individual information processor whose learning rate depends on how practice is organized and how feedback is scheduled. This tradition has generated robust findings on contextual interference (Shea and Morgan, 1979), self-controlled feedback (Chiviacowsky and Wulf, 2002), attentional focus (Wulf, 2013; Wulf et al., 1998), and feedback frequency/timing (Winstein and Schmidt, 1990; Janelle et al., 1997) that remain central to instructional design. Yet it rests on a premise that grows harder to defend as evidence accumulates: that the social environment in which practice unfolds is, at best, a background variable rather than a constitutive feature of learning.

Several convergent developments challenge this premise. In sport pedagogy, cooperative and dyadic practice formats have been shown to match or exceed individual practice on retention and transfer (Granados and Wulf, 2007; Shea et al., 1999). In developmental motor research, social-environmental factors such as parental involvement and peer modeling predict motor competence trajectories beyond task-specific practice (Adank et al., 2022; De Meester et al., 2021). In educational psychology, Self-Determination Theory (SDT) has amassed decades of evidence that the satisfaction of three basic psychological needs—autonomy, competence, and relatedness—underpins high-quality motivation, engagement, and learning across domains (Ryan and Deci, 2020; Vansteenkiste et al., 2020).

Within the motor learning literature, autonomy-related manipulations have received substantially more attention than relatedness, although recent meta-analytic evidence suggests these effects may be less robust than initially reported (McKay et al., 2025). Competence-supportive processes have also been examined, but often more indirectly and not always explicitly within an SDT framework. Self-controlled practice manipulations are typically interpreted as autonomy-supportive (Chiviacowsky and Wulf, 2002; Sanli et al., 2013), whereas informational feedback, optimal challenge, and expectancy-related manipulations are often treated as competence-relevant mechanisms within the broader motivational literature, including OPTIMAL theory (Wulf and Lewthwaite, 2016). Relatedness, however, remains conspicuously underexplored—addressed in only a handful of motor learning experiments (e.g., Chiviacowsky et al., 2016) despite being theorized as foundational to the other two needs (Baumeister and Leary, 1995). This asymmetry constitutes both an empirical gap and a conceptual blind spot.

To date, the most explicit attempt to incorporate motivational processes into motor learning theory is OPTIMAL theory, which emphasizes autonomy support, enhanced expectancies, and an external focus of attention in explaining performance and learning (Wulf and Lewthwaite, 2016). The SEML framework is intended as a complement to, rather than a replacement for, this line of work. My claim is therefore not that motivation has been absent from motor-learning theory, but that the relational and multilevel social conditions under which motivational supports are delivered and interpreted—such as trust, belonging, psychological safety, and cooperative structure—remain comparatively under-specified.

Against this background, the present article addresses two questions. First, what does the existing evidence suggest about the mechanisms through which social context may influence motor learning? Second, how can SDT—and relatedness in particular—provide a coherent theoretical account that yields testable predictions for future research? To address these questions, I synthesize converging evidence from multiple research traditions to develop the Socially Embedded Motor Learning (SEML) framework and to specify experimentally testable propositions.

The article is organized as follows. I first outline the theoretical background—SDT’s need structure and the contributions and limitations of the isolated-learner tradition. I then critically examine social contexts in motor learning at three levels: instructor–learner, peer, and broader socio-ecological. From this analysis, I derive the SEML framework with three core propositions and explicit boundary conditions. I close with methodological recommendations for testing the framework, practical implications, and a future research agenda.

### Theoretical background and critical problem framing

#### Self-determination theory: strengths and tensions

SDT posits that autonomy, competence, and relatedness are universal, innate psychological needs whose satisfaction promotes internalization, intrinsic motivation, and well-being (Deci and Ryan, 2000; Ryan and Deci, 2020; Vansteenkiste et al., 2020). In the present article, relatedness is defined as the learner’s subjective sense of being cared for, respected, accepted, and meaningfully connected to relevant others in the learning environment. This definition follows SDT in treating relatedness as a psychological need-satisfaction state rather than as the mere presence of other people, a structural property of the environment, or any single dyadic behavior (Baumeister and Leary, 1995; Ryan and Deci, 2020). Accordingly, I distinguish among three analytically separate levels: (a) social-context features (e.g., cooperative structures, instructor warmth, inclusive team norms), (b) relatedness-supportive cues or practices that communicate care, respect, and reliable inclusion, and (c) relatedness satisfaction, the learner’s experienced state of connection and belonging. Throughout the remainder of the manuscript, the term relatedness refers primarily to this third level unless otherwise specified.

However, a balanced appraisal must acknowledge tensions within and around SDT. The universality claim—that the three needs are invariant across cultures—has been debated. While cross-cultural studies generally support the functional significance of all three needs (Vansteenkiste et al., 2020), the relative salience of relatedness versus autonomy may shift considerably across collectivistic and individualistic cultural contexts, complicating predictions about which need-supportive interventions will be most effective in a given setting. Additionally, relatedness is the most loosely operationalized of the three needs: measures range from feeling connected to a specific partner, to sense of belonging in a group, to general social inclusion (Baumeister and Leary, 1995). This breadth offers flexibility but also creates measurement heterogeneity that limits comparability across studies. I return to these tensions when specifying boundary conditions for the proposed framework.

#### The isolated-learner paradigm: contributions and blind spots

The information-processing tradition in motor learning has clarified several robust regularities in the relation between practice structure and learning. For example, random practice often depresses immediate acquisition performance yet benefits retention and transfer relative to blocked practice (Shea and Morgan, 1979); an external focus typically yields more effective and efficient performance than an internal focus (Wulf et al., 1998); and reduced or self-controlled feedback can in some cases impair immediate performance while benefiting longer-term learning relative to more frequent externally imposed feedback (Winstein and Schmidt, 1990; Chiviacowsky and Wulf, 2002; Janelle et al., 1997). More recently, ecological dynamics approaches have enriched this picture by emphasizing perception-action coupling and environmental constraints (Araújo and Davids, 2011; Button et al., 2021; Renshaw and Chow, 2019). Yet even these extended frameworks tend to treat social interaction as one constraint among many rather than as a motivational system that regulates which task constraints the learner actually engages with.

Three specific blind spots warrant attention. First, many standard laboratory paradigms still examine one learner working primarily with an experimenter rather than with peers or teammates—a relational vacuum in which social cues are minimized by design; classic self-controlled feedback and videotape-replay paradigms are representative examples (Chiviacowsky and Wulf, 2002; Janelle et al., 1997). Second, the predominant dependent variables—acquisition curves, retention scores, and transfer tests—capture performance products but not the motivational and relational processes that sustain practice over weeks and months. Third, even when social variables are introduced (e.g., observation in dyadic practice, motor contagion between interaction partners; Ikegami and Ganesh, 2014), the theoretical mechanisms invoked are often cognitive (modeling, error detection) rather than explicitly relational (belonging, safety, trust). I therefore suggest not that motivation has been absent from motor-learning theory, but that the relational and multilevel social conditions through which motivational mechanisms operate remain comparatively under-specified.

### Critical analysis of social contexts in motor learning

#### Instructor–learner relationships: the relational foundation

A high-quality instructor–learner relationship—characterized by trust, respect, and care—provides relatedness-supportive cues that can foster relatedness satisfaction and, in turn, psychological safety: the shared belief that interpersonal risk-taking (e.g., admitting confusion, attempting difficult skills) is safe within the learning environment (Edmondson, 1999). Empirical evidence from self-controlled practice research suggests that when learners perceive the instructional context as supportive, they are more willing to treat feedback as informational rather than evaluative, to request challenging tasks, and to sustain practice through setbacks (Chiviacowsky and Wulf, 2002; Janelle et al., 1997). Although these studies did not directly manipulate relatedness satisfaction, the pattern is consistent with SDT’s prediction that relational quality conditions the motivational impact of instructional variables.

More direct evidence comes from autonomy-support intervention research in physical education. Cheon et al. (2018a), Cheon et al. (2018b), and Reeve and Cheon (2024) have shown across multiple trials that when teachers are trained in autonomy-supportive practices—which often include perspective-taking, acknowledgment of affect, and respectful communication—students report greater need satisfaction, engagement, and skill development. These interventions do not make autonomy and relatedness analytically identical; rather, they suggest that autonomy-supportive and relational cues frequently co-occur in applied settings, making their respective contributions difficult to disentangle. This overlap motivates the present framework’s emphasis on specifying how relatedness may condition the interpretation and impact of other need-supportive practices.

A candid assessment of the evidence, however, must note two limitations. First, the direct experimental evidence for relatedness effects on motor learning outcomes (as distinct from motivation or engagement) remains thin. Chiviacowsky et al. (2016) demonstrated that a brief relatedness manipulation enhanced motor performance, but the study used a single-session paradigm and a minimal social induction, leaving open questions about dose, duration, and ecological validity. Second, most supporting evidence is drawn from educational and sport psychology contexts where dependent variables emphasize motivation and engagement rather than the retention and transfer measures central to motor learning science. Bridging this gap requires purpose-built experiments that integrate motivational and motor learning methodologies.

#### Peer dynamics: cooperation, competition, and dyadic learning

Cooperative goal structures generally enhance both achievement and interpersonal attraction relative to competitive or individualistic structures, as established by decades of meta-analytic evidence (Johnson et al., 1981; Stanne et al., 1999). In motor learning specifically, cooperative formats can support relatedness satisfaction, sustain engagement, and facilitate shared problem-solving by distributing attentional demands across learners (Standage et al., 2025; Vazou et al., 2005). However, these benefits are neither automatic nor universal—they depend on task structure, group composition, and how cooperation is operationalized.

Dyadic practice represents a particularly well-studied cooperative format in motor learning. Alternating performer and observer roles has been shown to match or exceed solo practice on retention and transfer, even when each learner receives only half the physical practice trials (Granados and Wulf, 2007; Shea et al., 1999). The conventional explanation emphasizes cognitive mechanisms—observational learning, error detection, cognitive elaboration. I suggest this account is incomplete: dyads also create a minimal social unit that can support relatedness satisfaction (a partner who shares the task) and enable informational dialogue that reframes errors as shared learning opportunities. Consistent with this interpretation, Cheon et al. (2026) demonstrated that peer-directed agentic engagement—students’ constructive input into peer conversations to align interactions with their personal motivations—independently predicted gains in autonomy need satisfaction even after controlling for teacher-directed agentic engagement, suggesting that peer interactions serve a distinct motivational function beyond what instructor–learner dynamics alone can provide. Taken together, when dyadic practice is effective, it may be precisely because it co-activates social and cognitive learning mechanisms.

Competition, by contrast, presents a more complex picture. Murayama and Elliot’s (2012) meta-analysis of the competition–performance relationship revealed opposing processes: competition can enhance performance through increased arousal and effort while simultaneously undermining it through anxiety and ego-protective strategies. From an SDT perspective, the critical moderator is whether competitive structures preserve or erode relatedness. When competition is chronic, public, and zero-sum, it shifts attention toward ego protection and risk avoidance—thwarting both relatedness and the exploratory behaviors essential for deep learning. Conversely, when competition is embedded within cooperative structures (e.g., shared sub-goals, team-based scoring), it can clarify performance standards and elevate arousal without undermining belonging (Reeve and Cheon, 2024). This modulation is consistent with SDT’s prediction that identical structural features operate differently depending on the need-supportive or need-thwarting quality of the social climate.

Equity and inclusion further shape peer learning dynamics. Heterogeneous ability pairings can benefit novices through modeling and verbalization, but without structured role rotation, such pairings risk undermining competence and relatedness for both partners: novices may feel dependent, while experts may experience burden. Reciprocal task designs—where novices analyze and experts perform, then swap—maintain mutual agency and ensure that social exchanges remain informational rather than hierarchical (Granados and Wulf, 2007).

#### Broader socio-ecological contexts: parents, teams, and culture

The principles identified at the instructor and peer levels extend into broader socio-ecological systems. Parental autonomy support, co-activity, and positive attitudes toward physical activity predict children’s motor engagement and competence development (Adank et al., 2022; Clark et al., 2020). Within teams and classrooms, group cohesion, shared leadership, and a supportive motivational climate are linked to effort, information sharing, and coordinated action—conditions that plausibly create fertile ground for individual skill development (Carron et al., 2002; Fransen et al., 2020). At the same time, socio-cultural constraints—gendered sport norms, socioeconomic disparities, and resource inequities—shape access to relatedness-supportive learning environments (Clark et al., 2020; De Meester et al., 2021).

These levels—instructor, peer, family, team, and culture—are analytically separable yet likely interdependent. A coach who builds trust at the instructor level may enable cooperative practice structures at the peer level, which in turn operate within a team culture that values shared improvement, all under broader cultural norms about competition, gender, and authority. Indeed, autonomy-supportive teaching has been shown to produce spillover effects that extend beyond the immediate instructional context into students’ home environments (Cheon et al., 2024), illustrating how relational quality at one level can permeate adjacent levels. I therefore present this multilevel structure as a theoretically grounded synthesis of converging evidence rather than as an already established causal hierarchy.

### The socially embedded motor learning (SEML) framework

Synthesizing the preceding analysis, I propose the Socially Embedded Motor Learning (SEML) framework. The framework’s central claim is that social contexts—comprising instructor behaviors, peer structures, and socio-ecological factors—influence motor learning outcomes not merely by adding social input to an otherwise individual process, but by regulating the motivational quality with which learners engage practice. Relatedness occupies a specific functional role: it serves as a contextual catalyst that conditions how autonomy and competence support are interpreted and utilized by the learner.

To avoid conceptual ambiguity, I use four related but distinct terms throughout the framework. *Social context* refers to the structural and interpersonal features of the learning environment. *Relatedness support* refers to cues or arrangements that communicate care, respect, acceptance, and inclusion. *Relatedness satisfaction* refers to the learner’s subjective state of feeling connected and valued. A *relatedness-supportive context* is therefore one that is likely—but not guaranteed—to produce relatedness satisfaction. The propositions below concern relatedness satisfaction as the proximal learner-level mechanism.

#### Core propositions

Proposition 1: Amplification. When autonomy-supportive (e.g., choice, self-controlled feedback) and competence-supportive (e.g., informational feedback, optimal challenge) practices benefit motor learning, those effects are hypothesized to be amplified when learners experience high relatedness satisfaction. Inconsistency in the effects of these practices across studies may partly reflect uncontrolled variation in relational context: under conditions of high relatedness satisfaction, such supports may yield stronger and more reliable effects, whereas under conditions of low relatedness satisfaction, their benefits may be reduced or less consistent. The proposed mechanism is interpretive: when learners feel cared for, respected, and connected, they may be more likely to construe choice as genuine volition rather than abandonment, and feedback as useful information rather than evaluative judgment, thereby supporting intrinsic motivation and cognitive engagement (Cheon et al., 2018b; Patzak and Zhang, 2025).

Several lines of indirect evidence are consistent with this amplification hypothesis. In physical education, autonomy-supportive teaching appears to be most effective when embedded in a caring and relationally warm instructional climate—conditions likely to support relatedness satisfaction (Cheon et al., 2018a). Patzak and Zhang’s (2025) meta-analysis of autonomy support and structure provision likewise suggests that the effectiveness of these instructional strategies depends on the broader motivational climate, indicating that autonomy support may not operate in a relational vacuum. In the motor-learning literature, self-controlled practice paradigms have shown that learners in supportive instructional settings often request more challenging practice conditions and display better retention (Chiviacowsky and Wulf, 2002; Sanli et al., 2013), although these studies have not systematically isolated the relational component of the context from the autonomy manipulation itself.

An important alternative explanation must also be considered. It is possible that autonomy and competence support are sufficient conditions for learning gains and that relatedness merely contributes an additional motivational benefit without moderating how other supports are processed. Under this additive model, a 2 × 2 factorial design (high vs. low relatedness support × high vs. low autonomy support) would yield main effects without a significant interaction. The amplification hypothesis, by contrast, predicts a positive interaction such that autonomy-supportive practices should yield larger learning gains when relatedness satisfaction is concurrently high than when it is low, and this interaction should be evident on delayed retention and transfer tests rather than on acquisition performance alone. Distinguishing additive from multiplicative models is therefore the critical empirical test for Proposition 1.

Proposition 2: Safety-to-Exploration Pathway. Psychological safety is hypothesized to mediate the influence of relatedness satisfaction on adaptive learning behaviors. When learners feel connected, accepted, and valued, they may experience less evaluative threat, enabling greater error tolerance, help-seeking, and strategic movement variability—behaviors associated with deeper encoding and more robust motor memory (Edmondson, 1999; Wulf and Lewthwaite, 2016). This proposition specifies a serial pathway: social context → relatedness satisfaction → psychological safety → adaptive learning behaviors → enhanced retention/transfer.

The proposed pathway likely operates through several intermediate processes. First, by reducing evaluative threat, psychological safety may help learners maintain a more flexible regulatory state and broader attentional scope, making it easier to explore alternative coordination patterns rather than defaulting to safer, well-rehearsed movement solutions (Araújo and Davids, 2011; Button et al., 2021; Laborde et al., 2017). This possibility is broadly consistent with motor control research showing that anxiety and evaluative pressure can constrain movement organization, producing more rigid and conservative movement strategies (Higuchi et al., 2002; Nieuwenhuys and Oudejans, 2012). If psychological safety attenuates such threat, it may help preserve the exploratory movement variability that facilitates the discovery of efficient coordination solutions. Second, psychological safety may improve practice quality by increasing willingness to attempt difficult task variants, tolerate temporary performance decrements, and persist through error-rich practice phases—conditions likely to support deeper encoding and more robust retention, particularly for novel or complex tasks. This possibility may be especially relevant under practice conditions involving desirable difficulties, where immediate performance costs are tolerated in service of later learning (Magill and Anderson, 2021). Third, psychological safety may increase access to social informational resources by making learners more willing to ask questions, acknowledge confusion, and exchange feedback. In motor-learning contexts, where failure is visible and embodied, this reduction in self-presentational concern may be especially consequential (Edmondson, 1999). These mechanisms are theoretically plausible but remain only indirectly supported in motor-learning research.

Empirically, Proposition 2 predicts that psychological safety will mediate the relatedness–learning relationship when assessed at appropriately spaced time points, and that this mediation will be stronger for novel or complex tasks in which errors are more frequent and socially conspicuous.

Proposition 3: Cooperative-Structure Advantage. Dyadic and team-based practice formats may yield superior long-term motor-learning outcomes compared with purely individual or competitive conditions when two conditions are met: (a) the format reliably supports relatedness satisfaction (e.g., through structured interdependence and inclusion), and (b) it facilitates informational dialogue (e.g., through designated observation and feedback roles). When these conditions are met, cooperative structures may co-activate social and cognitive learning mechanisms (Johnson et al., 1981; Ntoumanis and Standage, 2009; Stanne et al., 1999).

Among the three propositions, some of the most direct evidence relevant to SEML comes from dyadic-practice studies, although it has usually been interpreted through a predominantly cognitive lens. Shea et al. (1999) and Granados and Wulf (2007) demonstrated that dyadic practice produced retention and transfer scores equal to or exceeding those of individual practice, despite each learner receiving only half the physical repetitions. The standard explanation invokes observational learning and cognitive elaboration: watching a partner attempt the task provides a second perspective on error detection and strategy evaluation. I argue that this account, while valuable, may be incomplete. Dyadic practice also creates a minimal social unit that can support relatedness satisfaction—a partner who shares the task goal, encounters similar challenges, and provides a non-evaluative audience for error. This relational dimension may help distinguish productive dyadic practice from passive video observation, which provides comparable visual information but lacks reciprocal social connection, although this distinction remains to be directly tested.

A critical caveat is that cooperation does not inherently support relatedness satisfaction. Unstructured group practice may generate social loafing, free-riding, or status hierarchies that undermine both competence and belonging. Vazou et al. (2005) documented how peer motivational climates in youth sport can be either mastery-oriented and inclusive or performance-oriented and exclusionary, with markedly different motivational consequences. The cooperative-structure advantage proposed here is therefore conditional: it depends on deliberate instructional design that ensures interdependent goals, equitable role distribution, and informational rather than evaluative peer feedback. When these structural conditions are absent, cooperative formats may offer no advantage—and may even impair learning relative to individual practice. This conditionality distinguishes Proposition 3 from a naive “social is better” claim and specifies the design features required for cooperative benefits to emerge.

#### Dynamic need interdependence

Although the propositions are presented separately, the three needs interact dynamically. Autonomy-supportive behaviors (providing choice, acknowledging perspective) often signal care and respect, thereby increasing relatedness satisfaction (Reeve, 2006; Reeve and Cheon, 2024). Conversely, competence feedback that is comparative or person-focused can erode belonging, dampening the motivational benefits typically associated with mastery cues. This bidirectionality implies that need co-satisfaction is not simply additive but potentially multiplicative: relatedness satisfaction conditions how autonomy and competence supports are processed, while autonomy and competence supports reciprocally reinforce relatedness satisfaction by conveying trust and credibility.

Concrete pedagogical scenarios illustrate these dynamics. Consider a coach who offers learners a choice of practice drills (autonomy support) but within a cold, impersonal instructional climate. The learner may interpret the choice not as empowerment but as indifference—“the coach does not care enough to guide me”—thereby undermining the intended autonomy benefit. The same choice, offered by a coach who has invested in rapport and demonstrates genuine interest in each learner’s development, is more likely to be experienced as trust and respect, deepening both autonomy and relatedness simultaneously. This interpretive dependency suggests that relatedness functions as a lens through which other motivational supports are appraised.

The reverse pathway is equally important. A physical therapist who provides technically excellent competence feedback (clear, specific, task-focused) but delivers it in a detached, clinical manner may inadvertently undermine relatedness, causing the patient to disengage from the rehabilitation program. In contrast, when competence feedback is embedded in a relational context that communicates care—“I notice you are working hard on this; here’s what I see that might help”—the same information strengthens both competence and relatedness satisfaction. These examples underscore that need satisfaction is not a matter of checking three independent boxes but of creating a coherent motivational climate in which the three needs reinforce one another synergistically.

#### Boundary conditions and moderators

A framework that claims social context matters must also specify when it matters less or differently. I identify four classes of moderators that constrain the generalizability of the SEML propositions and treat them as theoretically motivated boundary conditions rather than settled empirical conclusions.

Task complexity and type. For simple, well-defined tasks with clear performance criteria (e.g., a single key press in a reaction-time paradigm), the attentional demands of social interaction—monitoring a partner’s behavior, managing self-presentation, processing social cues—may compete with task-relevant processing and distract rather than facilitate (Murayama and Elliot, 2012). The social amplification predicted by Propositions 1 and 3 is expected to be strongest for complex, multi-degree-of-freedom tasks that require sustained practice, error correction, and strategic exploration—precisely the conditions where motivation matters most. In practical terms, tasks such as learning a tennis serve, acquiring a gymnastics skill, or rehabilitating gait after stroke involve prolonged error-correction cycles and high psychological demand, creating fertile conditions for relatedness effects. By contrast, laboratory tasks with minimal ecological complexity (e.g., linear positioning tasks) may show attenuated or null relatedness effects, which would not constitute evidence against the framework but rather confirm the predicted moderation. Researchers testing SEML propositions should therefore select tasks with sufficient complexity and ecological relevance to allow social mechanisms to operate meaningfully.

Expertise level. For novices facing high uncertainty, the safety-to-exploration pathway (Proposition 2) is expected to be particularly potent, as error tolerance and help-seeking are critical during early learning. For advanced learners who have already internalized task-relevant competence, the marginal contribution of relatedness support may diminish, and autonomy’s exploration benefits may predominate (Vansteenkiste et al., 2020). However, even expert performers may rely on relatedness support during periods of skill restructuring or performance slumps, suggesting a non-linear moderation.

Cultural context. SDT posits that all three needs are functionally important across cultures, although their expression and relative salience may vary. In more collectivistic or interdependence-oriented contexts, relatedness satisfaction may be more readily supported through group membership and shared identity, potentially strengthening Proposition 3 (cooperative advantage). In more individualistic contexts, autonomy support may exert more direct effects. Highly competitive scholastic or sport environments may also weaken relatedness effects unless cooperative structures are deliberately incorporated. Importantly, the forms through which relatedness support is communicated may themselves vary across cultures. In some East Asian educational and sport settings, for example, relatedness may be embedded within hierarchical relationships in which care is conveyed through invested guidance, high expectations, and sustained commitment to the learner’s development rather than primarily through egalitarian warmth more characteristic of many Western autonomy-supportive models (Ryan et al., 2025; Markus and Kitayama, 1991). Under such conditions, demanding instruction may sometimes be interpreted as evidence of relational commitment rather than simply as control, provided that learners also perceive respect, fairness, and benevolent intent. This suggests that the behavioral indicators of relatedness support may be culturally patterned even if the underlying psychological need is universal, and that cross-cultural tests of the SEML propositions should use culturally sensitive operationalizations of both relatedness support and relatedness satisfaction.

**Figure 1:**
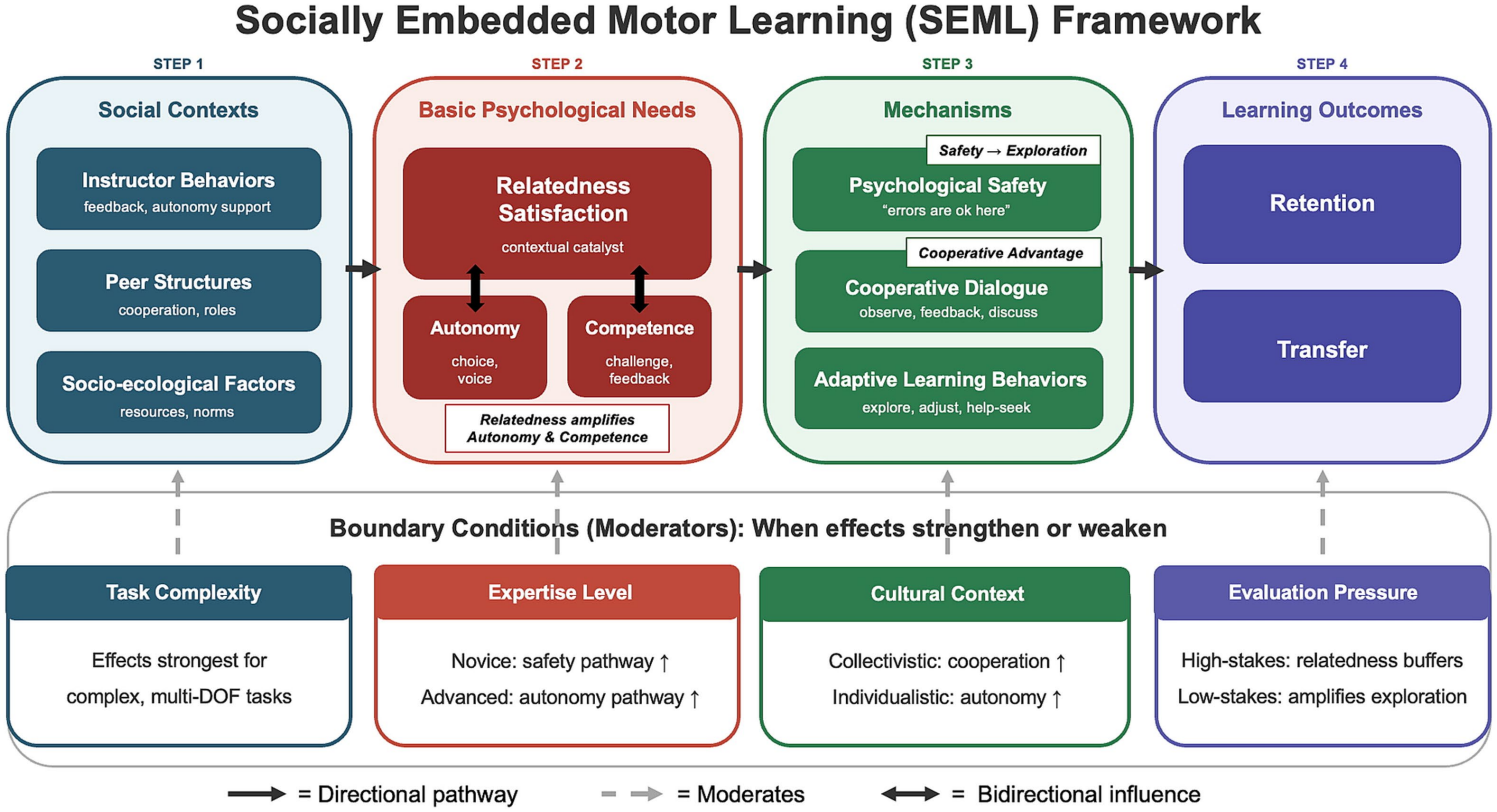
The socially embedded motor learning (SEML) framework. Social-context features (instructor behaviors, peer structures, and socio-ecological factors) are theorized to shape basic psychological need satisfaction. Relatedness satisfaction functions as a contextual catalyst that can strengthen the effects of autonomy- and competence-supportive practices (Proposition 1), foster psychological safety and adaptive learning behaviors (Proposition 2), and help make cooperative structures effective under specified conditions (Proposition 3). Solid arrows indicate primary directional pathways; dashed arrows indicate moderating influences; double-headed arrows indicate bidirectional need interdependence.

Evaluation context. High-stakes evaluation environments (formal testing, public competition) heighten evaluative threat and shift motivational orientation toward performance-avoidance goals. Under these conditions, relatedness support may serve a protective function—buffering against anxiety and maintaining engagement—rather than an amplification function. This suggests a qualitative shift in the mechanism linking relatedness to learning under different evaluation pressures. In low-stakes practice environments, relatedness amplifies exploration and risk-taking (the amplification and safety-to-exploration pathways dominate); in high-stakes environments, relatedness shifts toward an anxiety-buffering and persistence-maintenance role. Fletcher and Sarkar (2012) found that social support and positive relational bonds were among the most consistently reported protective factors in elite athletes’ resilience narratives, suggesting that under competitive pressure, relatedness serves as a psychological buffer rather than a performance enhancer. This dual functionality means that the observable behavioral signatures of relatedness support may differ across contexts: increased movement variability and exploration during practice, but increased emotional regulation and sustained effort during competition. The overall SEML framework, including the three propositions, their dynamic interdependence, and the relevant boundary conditions, is summarized in Figure 1.

### Methodological recommendations for testing the SEML framework

#### Design extensions

To test the SEML propositions, future studies should move beyond single-session, convenience-sampled paradigms toward multi-session interventions spanning several sessions or weeks in both applied and laboratory contexts. Designs that manipulate social-context variables—such as high- versus low-relatedness-support dyads or autonomy-supportive versus controlling instructors—while holding task difficulty constant would provide a stronger basis for causal inference about relational pathways. Crossed designs that factorially combine relatedness-support manipulations with autonomy- or competence-support manipulations are particularly well suited for testing Proposition 1’s amplification prediction. An interaction on delayed retention and transfer, especially when accompanied by corresponding changes in relatedness satisfaction, would provide strong evidence consistent with the hypothesized moderating role of relatedness. To test Proposition 2, studies should incorporate repeated assessments of psychological safety across multiple time points, enabling mediation analyses of the proposed safety-to-exploration pathway. Sequence-level manipulations (e.g., competition followed by cooperation versus the reverse) could further test whether the order in which social structures are introduced shapes subsequent safety and exploration. For Proposition 3, direct comparisons between structured cooperative formats (with designated roles and informational feedback) and unstructured group practice would help isolate the conditions under which cooperative advantages emerge.

#### Measurement strategy

Future work should combine validated need-satisfaction scales, such as the Basic Psychological Need Satisfaction and Frustration Scale (Chen et al., 2015), with context-adapted measures of psychological safety (e.g., based on Edmondson, 1999), motivational-regulation instruments, and delayed retention and transfer tests, which remain core dependent variables in motor learning. To preserve conceptual clarity, relatedness support should be assessed as a contextual feature or manipulation, whereas relatedness satisfaction should be measured as the learner’s subjective experience of connection and belonging. Because the BPNSFS relatedness subscale captures general need satisfaction with only a small number of items, researchers may benefit from supplementing it with context-specific relatedness measures that distinguish among different relational targets—for instance, felt connection to an instructor, a practice partner, or a team—and that capture both the presence and quality of belonging (e.g., perceived care and respect rather than mere social inclusion). For dyadic contexts, mutual partner ratings of relatedness would enable actor–partner interdependence analyses that separate within-person from cross-partner relational effects; in team settings, round-robin or sociometric approaches may further clarify the distribution of relatedness within the learning environment. Complementary behavioral coding should capture help-seeking and inquiry, peer-feedback quality (informational versus evaluative), instructor micro-behaviors (autonomy−/relatedness-supportive versus thwarting), and affective markers such as shared positive affect during error exploration. This multi-method strategy would allow psychological constructs and observable learning processes to be assessed jointly.

#### Analytic strategies

The nested and dynamic nature of socially embedded learning calls for analytic approaches that can model both interdependence and change over time. Multilevel models can account for learners nested within dyads or teams, while multilevel moderation and moderated-mediation models are especially appropriate for testing the interaction and pathway claims embedded in Propositions 1 and 2. Random-intercept cross-lagged panel models (RI-CLPM; Hamaker et al., 2015) may be used to examine reciprocal longitudinal relations among social climate, relatedness satisfaction, psychological safety, motivation, and performance. In dyadic contexts, actor–partner interdependence models (APIM; Kenny et al., 2006) can distinguish within-person from cross-partner influences.

#### Neuroscience and computational directions

The integration of neuroscience and psychophysiology offers a promising, though still speculative, direction for future SEML research. Combining EEG, fMRI, or heart rate variability (HRV) measures with cooperative and competitive task contexts could help clarify how social interaction shapes reward processing, attention, and memory consolidation during motor learning (Ikegami and Ganesh, 2014; Laborde et al., 2017). HRV, in particular, may serve as a real-time index of regulatory flexibility under supportive versus controlling instructional cues. Emerging computational models of social cognition (Lockwood and Klein-Flügge, 2021) could also be extended to incorporate social reward functions representing relatedness satisfaction, thereby simulating how motivational states shape exploration–exploitation trade-offs during skill acquisition. Because these directions remain largely untested in motor-learning contexts, they should be pursued with appropriate interpretive caution.

## Discussion

### Theoretical contributions

This article makes three interconnected contributions. First, it identifies relatedness as a comparatively neglected but functionally central variable in motor learning—one that operates not as an independent additive factor but as a catalyst conditioning how other forms of instructional support are experienced. This reframes the social dimension of motor learning from a peripheral concern to a design variable with greater mechanistic specificity.

Second, by formulating three testable propositions and specifying boundary conditions, the SEML framework offers a bridge between SDT’s psychological principles and the experimental methodology of motor learning science. Existing work—including OPTIMAL-oriented research—has productively manipulated autonomy-supportive and expectancy-related variables and, in some cases, measured motivational states alongside performance. My argument is narrower: the relational causal architecture linking social context, relatedness satisfaction, psychological safety, and motor-learning outcomes remains comparatively less specified. The propositions advanced here provide a starting point for experiments that can test those specific relational-motivational pathways.

Third, the article advances a meta-theoretical argument for integrating motor control and motivational psychology, disciplines that have developed largely in isolation despite addressing overlapping phenomena. The SEML framework positions motor learning at the intersection of motivational psychology, social neuroscience, and pedagogy, suggesting that the performer is not merely an information-processing system but a socially embedded agent whose motor adaptation is co-regulated through interpersonal dynamics (Lockwood and Klein-Flügge, 2021; Wulf and Lewthwaite, 2016).

In this sense, the SEML framework is intended to complement rather than replace OPTIMAL theory (Wulf and Lewthwaite, 2016). Whereas OPTIMAL offers a parsimonious account of individual-level motivational and attentional optimization, SEML focuses more explicitly on the social-relational conditions—particularly relatedness satisfaction and psychological safety—that shape how autonomy- and competence-supportive practices are interpreted and translated into learning behavior.

### Cross-context synthesis

The framework helps reconcile findings that can appear discrepant across laboratory and field settings. Under laboratory conditions that heighten evaluative threat, relatedness and psychological safety may disproportionately influence the learner’s willingness to attempt high-variance practice strategies. In team sport settings, cooperative micro-structures (e.g., shared constraints in small-sided games) may help translate autonomy into strategic exploration without the social costs that can accompany individual risk-taking in a more evaluative climate (Button et al., 2021; Renshaw and Chow, 2019). These correspondences suggest that task constraints and social climate jointly determine whether movement variability is experienced as adaptive exploration or as threatening error.

### Limitations

Several limitations warrant acknowledgment. The theoretical propositions advanced here are grounded in a purposive synthesis of existing evidence that prioritizes analytical depth and integrative argumentation over exhaustive coverage (Snyder, 2019). They are, by design, arguments to be tested rather than empirically settled conclusions.

The most significant limitation is the limited direct experimental evidence for relatedness effects on motor-learning outcomes. The majority of supporting evidence is drawn from sport psychology and educational research, where dependent variables often emphasize motivation, engagement, and well-being rather than the delayed retention and transfer measures that are commonly treated as core indicators of motor learning. I have been transparent about this evidentiary gap throughout the article and present the SEML framework as a research-generative model that specifies where and how this gap can be addressed, rather than as a summary of established findings.

Additionally, the article relies heavily on SDT as its theoretical lens, which necessarily foregrounds certain motivational mechanisms while backgrounding others (e.g., achievement goal theory, expectancy-value models). Future work integrating multiple motivational frameworks could provide a more comprehensive account. Finally, the cultural generalizability of the framework requires empirical testing, particularly in non-Western educational and sporting contexts where relational dynamics and authority structures may differ substantially.

### Practical implications

The SEML framework yields actionable recommendations for professionals who design and deliver motor learning experiences—coaches, physical educators, and rehabilitation specialists.

#### Build relational foundations before technical instruction

Rapport-building is not a preliminary nicety but a foundational instructional condition that shapes the effectiveness of subsequent practice design. Practical strategies include beginning sessions with brief reflective dialogues (e.g., “What’s one thing you tried and learned from since last time?”), using learners’ names, acknowledging effort and emotional states, and engaging in collaborative goal-setting. These micro-behaviors communicate care and can foster the psychological safety needed for learners to embrace challenge (Edmondson, 1999; Fletcher and Sarkar, 2012).

#### Engineer cooperative structures intentionally

Structured dyads and small-group activities with interdependent goals can strengthen relatedness satisfaction and enhance persistence. When competition is present, it can be framed as a shared challenge within psychologically safe teams rather than as zero-sum social comparison. Rotating roles—such as performer, observer, and analyst—can help ensure that peer interaction is both relationally supportive and informationally rich.

#### Frame feedback as informational and feed-forward

Rather than emphasizing correctness or error counts, instructors can promote learner autonomy and strategic reflection with questions such as “What did you notice?” and “What will you adjust next?” This feed-forward orientation shifts attention from external judgment to self-regulation, reinforcing both autonomy and competence (Haerens et al., 2018).

#### Design environmental cues that signal safety and growth

Progress-oriented displays, shared learning goals, and normalized error discussion (e.g., “error-of-the-day” routines) can create an ambient climate of psychological safety. Importantly, these motivational supports may be especially valuable during competitive periods, when cooperative rehearsals or debriefs can help emphasize mutual learning before formal rankings are introduced (Fransen et al., 2020).

Collectively, these strategies treat social architecture as a core design variable in motor learning environments. Programs integrating autonomy- and relatedness-supportive pedagogy have shown improvements in engagement and, in some contexts, performance in physical education and teacher education settings (Behzadnia et al., 2020; Patzak and Zhang, 2025), offering preliminary practical support for this broader approach.

## Conclusion

This article has argued that motor learning cannot be fully characterized as an intrapersonal optimization problem. Evidence across instructor, peer, and socio-ecological contexts suggests that social relationships and motivational climates help regulate learner engagement, risk-taking, and persistence—the very behaviors that influence whether practice translates into durable skill. By centering relatedness within SDT’s need framework, the SEML model offers a specific and testable account of how social context enters the learning equation: not as background noise, but as a catalyst that shapes how learners interpret, engage with, and benefit from instructional support.

The framework’s three propositions—amplification, safety-to-exploration, and cooperative-structure advantage—together with their specified boundary conditions, provide a concrete research agenda. I have been candid about the framework’s current evidentiary limitations: the direct experimental base in motor learning remains limited, and much of the supporting evidence is indirect. This transparency is intentional. The SEML framework is offered not as a summary of established findings, but as a generative model that specifies what should be tested, how, and under what conditions.

For practitioners, the core message is that relationships and motivational climates deserve the same design attention as practice schedules and feedback protocols. For researchers, the invitation is not to displace existing motivational accounts such as OPTIMAL theory, but to extend them by treating the social and relational architecture of practice as a theoretically motivated, experimentally manipulable, and rigorously measurable component of motor learning.

## Data Availability

No datasets were generated or analyzed for this study. Further inquiries can be directed to the corresponding author.

## References

[ref1] Adank C. Brown A. Verswijveren S. J. J. M. (2022). Parent involvement in motor skill interventions: a systematic review. Early Years 44, 400–420. doi: 10.1080/09575146.2022.2034174

[ref2] Araújo D. Davids K. (2011). What exactly is acquired during skill acquisition? A problem of ecological validity. Hum. Mov. Sci. 30, 869–888. doi: 10.1016/j.humov.2011.06.002, 21802756 PMC3183280

[ref3] Baumeister R. F. Leary M. R. (1995). The need to belong: desire for interpersonal attachments as a fundamental human motivation. Psychol. Bull. 117, 497–529. doi: 10.1037/0033-2909.117.3.497, 7777651

[ref4] Behzadnia B. Adachi P. J. C. Deci E. L. (2020). Autonomy-supportive teaching and students' game performance. J. Teach. Phys. Educ. 40, 438–446. doi: 10.1123/jtpe.2019-0253

[ref5] Button C. Seifert L. Chow J. Y. Araújo D. Davids K. (2021). Dynamics of Skill Acquisition: An Ecological Dynamics Approach. Human Kinetics Publishers.

[ref6] Carron A. V. Bray S. R. Eys M. A. (2002). Team cohesion and success in sport. J. Sports Sci. 20, 119–126. doi: 10.1080/026404102317200828, 11811568

[ref7] Chen B. Vansteenkiste M. Beyers W. Boone L. Deci E. L. Van der Kaap-Deeder J. . (2015). Basic psychological need satisfaction, need frustration, and need strength across four cultures. Motiv. Emot. 39, 216–236. doi: 10.1007/s11031-014-9450-1

[ref8] Cheon S. H. Reeve J. Huang D. Patall E. A. Im C.-H. Kim U. N. (2026). Greater autonomy need satisfaction with a little help from my friends: a randomized control trial to increase peer-directed agentic engagement. Contemp. Educ. Psychol. 85:102447. doi: 10.1016/j.cedpsych.2026.102447

[ref9] Cheon S. H. Reeve J. Jang H. R. (2018a). Autonomy-supportive teaching in physical education: how self-determined motivation affects learning and engagement. Contemp. Educ. Psychol. 55, 211–222. doi: 10.1016/j.cedpsych.2018.09.002

[ref10] Cheon S. H. Reeve J. Jang H.-R. Pink M. A. Song Y.-G. Im C.-H. (2024). Autonomy-supportive teaching leads to autonomy-supportive parenting: a teacher-to-parent relationship spillover effect. Teach. Teach. Educ. 144:104548. doi: 10.1016/j.tate.2024.104548

[ref11] Cheon S. H. Reeve J. Lee Y. Lee J. W. (2018b). Why autonomy-supportive interventions work: explaining the professional development of teachers' motivating style. Teach. Teach. Educ. 69, 43–51. doi: 10.1016/j.tate.2017.09.022

[ref12] Chiviacowsky S. Wulf G. (2002). Self-controlled feedback and learning. Res. Q. Exerc. Sport 73, 408–415. doi: 10.1080/02701367.2002.10609040, 12495242

[ref13] Chiviacowsky S. Wulf G. Lewthwaite R. (2016). Relatedness support enhances motor learning. Sport Exerc. Perform. Psychol. 5, 72–82. doi: 10.1037/spy0000052

[ref14] Clark C. C. Pearsall-Jones J. G. Piek J. P. (2020). Socioeconomic status and motor development: a systematic review. Child Care Health Dev. 46, 265–277. doi: 10.1111/cch.12748, 31978249

[ref15] De Meester A. Barnett L. M. Brian A. Stodden D. F. (2021). The relationship between social environmental factors and motor performance in 3- to 12-year-old typically developing children: a systematic review. J. Sports Sci. 39, 1765–1778. doi: 10.1080/02640414.2021.1899999 PMC8306533 34299967

[ref16] Deci E. L. Ryan R. M. (2000). The "what" and "why" of goal pursuits: human needs and the self-determination of behavior. Psychol. Inq. 11, 227–268. doi: 10.1207/S15327965PLI1104_01

[ref17] Edmondson A. (1999). Psychological safety and learning behavior in teams. Admin. Sci. Q. 44, 350–383. doi: 10.2307/2666999

[ref18] Fletcher D. Sarkar M. (2012). A grounded theory of psychological resilience in Olympic champions. Psychol. Sport Exerc. 13, 669–678. doi: 10.1016/j.psychsport.2012.04.007

[ref19] Fransen K. Vanbeselaere N. De Cuyper B. Vande Broek G. Boen F. (2020). The impact of shared leadership on team functioning and performance in sport. J. Sports Sci. 38, 2432–2440. doi: 10.1080/02640414.2020.1777109

[ref20] Granados C. Wulf G. (2007). Dyad practice: observation and dialogue contributions. Res. Q. Exerc. Sport 78, 197–203. doi: 10.1080/02701367.2007.10599419, 17679493

[ref21] Haerens L. Vansteenkiste M. Aelterman N. (2018). Promoting high-quality motivation in physical education: from theory to practice. J. Teach. Phys. Educ. 37, 360–374. doi: 10.1123/jtpe.2018-0182

[ref22] Hamaker E. L. Kuiper R. M. Grasman R. P. P. P. (2015). A critique of the cross-lagged panel model. Psychol. Methods 20, 102–116. doi: 10.1037/a0038889, 25822208

[ref23] Higuchi T. Imanaka K. Hatayama T. (2002). Freezing degrees of freedom under stress: kinematic evidence of constrained movement strategies. Hum. Mov. Sci. 21, 831–846. doi: 10.1016/S0167-9457(02)00174-4, 12620722

[ref24] Ikegami T. Ganesh G. (2014). Motor contagion and communication partners. PLoS One 9:e106172. doi: 10.1371/journal.pone.0106172, 25153990 PMC4143359

[ref25] Janelle C. M. Barba D. A. Frehlich S. G. Tennant L. K. Cauraugh J. H. (1997). Videotape replay in self-controlled learning. Res. Q. Exerc. Sport 68, 269–279. doi: 10.1080/02701367.1997.10608008 9421839

[ref26] Johnson D. W. Maruyama G. Johnson R. Nelson D. Skon L. (1981). Cooperative vs. competitive goal structures: a meta-analysis. Psychol. Bull. 89, 47–62. doi: 10.1037/0033-2909.89.1.47

[ref27] Kenny D. A. Kashy D. A. Cook W. L. (2006). Dyadic Data Analysis. New York: Guilford Press.

[ref28] Laborde S. Mosley E. Thayer J. F. (2017). Heart rate variability and cardiac vagal tone in psychophysiological research—recommendations for experiment planning, data analysis, and data reporting. Front. Psychol. 8:213. doi: 10.3389/fpsyg.2017.00213, 28265249 PMC5316555

[ref29] Lockwood P. L. Klein-Flügge M. C. (2021). Computational modelling of social cognition and behaviour. Nat. Rev. Neurosci. 22, 770–788. doi: 10.1038/s41583-021-00504-3 PMC8343561 32232358

[ref30] Magill R. A. Anderson D. I. (2021). Motor Learning and Control: Concepts and Applications. 12th Edn New York: McGraw-Hill.

[ref31] Markus H. R. Kitayama S. (1991). Culture and the self: implications for cognition, emotion, and motivation. Psychol. Rev. 98, 224–253. doi: 10.1037/0033-295X.98.2.224

[ref32] McKay B. Bacelar M. F. B. Parma J. O. Miller M. W. Carter M. J. (2025). The combination of reporting bias and underpowered study designs has substantially exaggerated the motor learning benefits of self-controlled practice and enhanced expectancies: a meta-analysis. Int. Rev. Sport Exerc. Psychol. 18, 242–262. doi: 10.1080/1750984X.2023.2207255

[ref33] Murayama K. Elliot A. J. (2012). The competition–performance relation: a meta-analytic review and test of the opposing processes model of competition and performance. Psychol. Bull. 138, 1035–1070. doi: 10.1037/a0028324, 23088570

[ref34] Nieuwenhuys A. Oudejans R. R. D. (2012). Anxiety and perceptual-motor performance: toward an integrated model of concepts, mechanisms, and processes. Psychol. Res. 76, 747–759. doi: 10.1007/s00426-011-0384-x, 22038472 PMC3470682

[ref35] Ntoumanis N. Standage M. (2009). Motivation in physical education classes: a self-determination theory perspective. Theory Res. Educ. 7, 194–202. doi: 10.1177/1477878509104324

[ref36] Patzak A. Zhang X. (2025). Blending teacher autonomy support and provision of structure in the classroom for optimal motivation: a systematic review and meta-analysis. Educ. Psychol. Rev. 37:17. doi: 10.1007/s10648-025-09994-2

[ref37] Reeve J. (2006). Teachers as facilitators: what autonomy-supportive teachers do. Elem. Sch. J. 106, 225–236. doi: 10.1086/501484

[ref38] Reeve J. Cheon S. H. (2024). Learning how to become an autonomy-supportive teacher begins with perspective taking: a randomized control trial and model test. Teach. Teach. Educ. 148:104702. doi: 10.1016/j.tate.2024.104702

[ref39] Renshaw I. Chow J. Y. (2019). A constraint-led approach to sport and physical education pedagogy. Phys. Educ. Sport Pedagogy 24, 103–116. doi: 10.1080/17408989.2018.1552676

[ref40] Ryan R. M. Deci E. L. (2020). Self-Determination Theory: Basic Psychological Needs in Motivation, Development, and Wellness. 2nd Edn New York: Guilford Press.

[ref41] Ryan R. M. Jang H. Wang J. C. K. Matos L. Gordeeva T. Kaplan H. . (2025). Variations in need supports in education as a function of cultural and economic factors: perspectives from self-determination theory. Educ. Psychol. Rev. 37:118. doi: 10.1007/s10648-025-10088-2

[ref42] Sanli E. A. Patterson J. T. Bray S. R. Lee T. D. (2013). Understanding self-controlled motor learning protocols through the self-determination theory. Front. Psychol. 3:611. doi: 10.3389/fpsyg.2012.00611, 23430980 PMC3576889

[ref43] Shea J. B. Morgan R. L. (1979). Contextual interference effects on the acquisition, retention, and transfer of a motor skill. J. Exp. Psychol. Hum. Learn. Mem. 5, 179–187. doi: 10.1037/0278-7393.5.2.179

[ref44] Shea C. H. Wulf G. Whitacre C. A. (1999). Enhancing efficiency via dyad training. J. Mot. Behav. 31, 119–125. doi: 10.1080/00222899909600986, 11177626

[ref45] Snyder H. (2019). Literature review as a research methodology: an overview and guidelines. J. Bus. Res. 104, 333–339. doi: 10.1016/j.jbusres.2019.07.039

[ref46] Standage M. Ryan R. M. Curran T. (2025). “Self-determination theory applied to physical education: the role of self-regulatory processes in facilitating high-quality student motivation, engagement, and well-being,” in in García-González, L., De Cocker, K., and González-Cutre, D. (eds.), Motivation in Physical Education, (Cham: Springer Nature), 29–51.

[ref47] Stanne M. B. Johnson D. W. Johnson R. T. (1999). Does competition enhance or inhibit motor performance? Psychol. Bull. 125, 133–154. doi: 10.1037/0033-2909.125.2.133, 9990847

[ref48] Vansteenkiste M. Ryan R. M. Soenens B. (2020). Basic psychological need theory: advancements, critical themes, and future directions. Motiv. Emot. 44, 1–31. doi: 10.1007/s11031-019-09818-1

[ref49] Vazou S. Ntoumanis N. Duda J. L. (2005). Peer motivational climate in youth sport: a qualitative inquiry. Psychol. Sport Exerc. 6, 497–516. doi: 10.1016/j.psychsport.2004.03.005

[ref50] Winstein C. J. Schmidt R. A. (1990). Reduced frequency of knowledge of results enhances motor skill learning. J. Exp. Psychol. Learn. Mem. Cogn. 16, 677–691. doi: 10.1037/0278-7393.16.4.677

[ref51] Wulf G. (2013). Attentional focus and motor learning: a review of 15 years. Int. Rev. Sport Exerc. Psychol. 6, 77–104. doi: 10.1080/1750984X.2012.723728

[ref52] Wulf G. Höß M. Prinz W. (1998). Instructions for motor learning: differential effects of internal versus external focus of attention. J. Mot. Behav. 30, 169–179. doi: 10.1080/00222899809601334 20037032

[ref53] Wulf G. Lewthwaite R. (2016). Optimizing performance through intrinsic motivation and attention for learning: the OPTIMAL theory of motor learning. Psychon. Bull. Rev. 23, 1382–1414. doi: 10.3758/s13423-015-0999-9, 26833314

